# Socioeconomic Inequalities in pediatric Metabolic Syndrome: mediation by parental health literacy

**DOI:** 10.1093/eurpub/ckac131.191

**Published:** 2022-10-25

**Authors:** A Lepe, MLA de Kroon, SA Reijneveld, AF de Winter

**Affiliations:** 1 Health Sciences, UMC Groningen, Groningen, Netherlands; 2 Public Health and Primary Care, KU Leuven, Leuven, Belgium

## Abstract

**Background:**

Parental health literacy may explain the relationship between parental socioeconomic status (SES) and pediatric metabolic syndrome (MetS). For this reason, we assessed to what extent parental health literacy mediates the relationships between parental SES and pediatric MetS.

**Methods:**

We used data from the prospective multigenerational Dutch Lifelines Cohort Study. Our sample consisted of 6,683 children with an average follow-up of 36.2 months (SD 9.3) and a mean baseline age of 12.8 years (SD 2.6). We used natural effects models to assess the natural direct, natural indirect, and total effects of parental SES on MetS.

**Results:**

On average, an additional four years of parental education, e.g. university instead of secondary school, would lead to cMetS scores that were 0.499 (95% confidence interval (CI) 0.364; 0.635) units lower, which is a small effect (d 0.18). If parental income and occupational level were one standard deviation higher, on average cMetS scores were 0.136 (95%CI 0.052; 0.219) and 0.196 (95%CI 0.108; 0.284) units lower, respectively; these are both small effects (d 0.05 and 0.07, respectively). Parental health literacy partially mediated these pathways; it accounted for 6.7% (education), 11.8% (income), and 8.3% (occupation) of the total effect of parental SES on pediatric MetS.

**Conclusions:**

Socioeconomic differences in pediatric MetS are relatively small, the largest being by parental education. Improving parental health literacy may reduce these inequalities. Further research is needed into the mediating role of parental health literacy on other socioeconomic health inequalities in children.

**Key messages:**

• Parental socioeconomic status (SES) has a small inverse relationship with pediatric metabolic syndrome (MetS), which is partially mediated by parental health literacy.

• Targeting parental health literacy may reduce inequalities in pediatric MetS. It may also influence other pediatric socioeconomic health inequalities, but further research is needed.

